# Targeting the microbiome: from probiotics to fecal microbiota transplantation

**DOI:** 10.1186/s13073-018-0592-8

**Published:** 2018-10-30

**Authors:** Alexander Khoruts

**Affiliations:** Department of Medicine, Division of Gastroenterology, Hepatology, and Nutrition, Center for Immunology, and BioTechnology Institute, University of Minnesota, Wallin Medical Biosciences Building, 2101 6th Street South East, Room 3-184, Minneapolis, MN 55414 USA

**Keywords:** Colonization resistance, Fecal microbiota transplantation, Microbiome, Microbiota, Probiotics

## Abstract

The modern techniques of microbiome science can be applied to the development and evaluation of all microbiota-directed products, including probiotics and fecal microbiota transplantation.

## Probiotics through the lens of microbial ecology

The advent of high-throughput sequencing technologies and advanced computational techniques has enabled a paradigm shift in how scientists view and study the microbial world. The term “microbiome” is now part of the common vernacular, and there is an increasing recognition that various environmental inputs such as antibiotics and nutrition may impact health and disease via their effects on the commensal intestinal microbiota. Physicians commonly prescribe probiotics, often at patients’ requests and often as an antidote to the potential harms of antibiotics [1]. Many probiotic products claim to promote and restore microbial balance in the body. However, pre-market regulatory approval for these claims is not required, at least in the USA, where probiotics are generally classified as dietary supplements. Current technologies that enable comprehensive characterization of microbial communities are now beginning to be applied to the evaluation of microbial products. This comment will consider the cases of probiotics and fecal microbiota transplantation (FMT), both of which target the intestinal microbiota but operate within very different regulatory frameworks.

## Investigating the effects of probiotics and FMT on the gut microbiota

Many probiotic products claim to promote “healthy” or “balanced” gut flora. But what does that mean? Even now, we are still only beginning to understand the ranges of normal microbiome composition in healthy individuals and its alterations in various disease states. In two recent papers, Elinav and colleagues attempted to tackle this question [2, 3]. They used modern techniques of microbiome science, which enable characterization of microbial community composition and gene content, to test the effects of probiotic consumption on intestinal microbiota in the absence of or following antibiotic treatment in mice and human volunteers.

In one set of experiments [2], a commercial preparation (Bio-25) containing 11 species of common probiotic bacteria (an assortment of *Lactobacilli*, *Bifidobacteria*, *Lactococcus lactis*, and *Streptococcus thermophilus*) was fed daily to healthy volunteers for 28 days. The fed probiotic bacteria were detected in stool samples of all participants as long as they were consuming the product, but were found in the colonic mucosa in only some participants. This transient engraftment was dependent on the microbiome composition of the participants, and transfer of human microbiota into germ-free mice replicated the permissive versus resistant phenotypes in the recipient animals.

In another set of experiments [3], the investigators tested the effects of the same probiotic preparation on microbiome recovery after a 1-week course of commonly used antibiotics (ciprofloxacin and metronidazole). Not surprisingly, antibiotics had a profound effect on the indigenous microbiota of experimental mice and human participants. The antibiotic-induced disruption of intestinal microbiota also allowed for some engraftment of probiotic bacteria into mice (no engraftment was seen in mice without antibiotic conditioning) and markedly enhanced colonization by probiotic bacteria in human participants, although still with considerable inter-individual differences. Administration of probiotics delayed microbiome recovery to a pre-antibiotic state in both mice and humans. In contrast, administration of autologous FMT accelerated microbiome recovery to a pre-antibiotic state.

The effects of antibiotic-induced microbiota disturbance were also evident in the functional potential of the microbiota and mucosal host gene expression. Probiotics delayed normalization, whereas autologous FMTs accelerated normalization. One of the functions of the indigenous microbiota that was disrupted by antibiotics and delayed in recovery by probiotics was secondary bile acid metabolism, which plays an important role in protection against *Clostridium difficile* infection, a common complication of antibiotic therapies [4].

## Therapeutic targeting of the microbiome

The notion of “good microbes” that may benefit host health originated in the early twentieth century when live microbes contained in fermented foods were thought to mitigate the postulated toxic effects of the commensal gut flora [5]. Early use of the term “probiotics” in the 1970s was linked to the concept of promoting “intestinal microbial balance,” even though it was unclear how such “balance” was to be measured. Since then, interest in probiotics has grown substantially. The most widely accepted scientific definition of probiotics is from the 2001 Expert Consultation for the Food and Agriculture Organization (FAO) of the United Nations and the World Health Organization (WHO), which states that probiotics are “live microorganisms which when administered in adequate amounts confer a health benefit on the host.” Multiple mechanisms have been described using in vitro and animal models for how probiotics may mediate such beneficial effects, including direct inhibition of pathogens or their products, mucosal immunomodulation, enhanced gut barrier function, and others. However, despite promising pre-clinical data, strong evidence for benefit in treatment of any disease condition in humans has not yet been established.

FMT in Western medicine also has its roots dating back to the introduction of antibiotics [6]. However, it has come to be widely used only relatively recently as a therapeutic strategy to repair the microbiota in patients with recurrent *Clostridium difficile* infection. The intestinal microbiota of these patients sustains severe damage from multiple rounds of antibiotic treatment. Transferring the entire donor microbiota inoculum achieves a donor-like microbiota composition with normalization of its functionality [7]. This approach recognizes microbiota as a complex unit and uses an organ or tissue “transplant” therapeutic paradigm.

The methodology for separating microbiota from stool and its cryopreservation allows for rigorous selection of donors and testing for blood and enteric pathogens [8]. The same methodology can also be used for autologous FMT where a patient’s own microbiota can be saved prior to its disruption and re-introduction later to recover the pre-treatment microbiota composition. Such autologous FMT was tested recently by Taur and colleagues [9] against a placebo in patients undergoing allogeneic hematopoietic stem cell transplantation, a treatment associated with severe gut damage, suppression of the immune system, and heavy exposure to antibiotics. Not surprisingly, autologous FMT, but not the placebo, was associated with an increase in microbial diversity and re-establishment of pre-antibiotic microbiota composition. Although the clinical trial is still in progress, it is hoped that prompt recovery of the normal microbiota functionality will translate into fewer bloodstream and enteric infections and mitigate the potential for development of graft-versus-host disease.

## Drug versus dietary supplement versus transplant

The FAO/WHO Working Group on probiotics produced guidelines for potential regulators of these products. These guidelines recognized that live probiotic microbes could have deleterious effects in some patients, especially those with a compromised immune system; their effects may be strain-dependent; labeling should include expected numbers of live microorganisms at the time of product expiration; and large, randomized, placebo-controlled clinical trials should be conducted for products intended to treat disease conditions. Only the last point is reflected in the current laws in the USA where any agent that treats, mitigates, prevents, or cures a disease is classified as a drug and needs to be validated by well-executed clinical trials. It is not surprising, therefore, that most clinical trials with probiotics are low quality and consequently the numerous meta-analyses remain contradictory or inconclusive. Thus, there is scant evidence on the effects of the currently available probiotic products, which given their common use need more rigorous investigations. Furthermore, in a recent systematic review [10], only a meager 2% of randomized clinical trials with probiotics adequately reported key safety components. The problem is further compounded by the multitude of preparations, lot-to-lot variability, and labeling inconsistencies [11].

In contrast, despite its name, FMT is classified as a drug in the USA. This is the case even for autologous FMT. Currently, treatment of *C. difficile* infection that cannot be cured with antibiotics can be treated with FMT under the FDA enforcement discretion policy without agency approval. However, this is a temporary policy until an effective drug product is approved to fill the unmet need. It remains to be seen whether such a drug product will be a defined microbial product or an FMT preparation. It is also important that any microbial therapeutic that is ultimately approved for a specific indication, such as *C. difficile* infection, undergoes rigorous trials and is not assumed to be also useful for treatment of other conditions such as inflammatory bowel disease, diabetes, or autism.

## Microbiome science in evaluation of microbial therapeutics

Although our understanding of the microbiome remains limited, we have entered the age when microbiome science can and should be incorporated into the evaluation of microbial products. Elinav and colleagues [2, 3] did not evaluate the clinical effects of probiotics, but they were able to demonstrate an impact on post-antibiotic recovery of the indigenous microbiota with the specific product they tested. Their studies also showed marked variability among healthy participants in the mucosal engraftment of probiotic bacteria, which was dependent on the individual participants’ microbiomes. Host microbiome variability was not considered by the FAO/WHO Working Group, but should be incorporated into future clinical trial designs of microbial therapeutics. Clinical outcomes of microbiota-directed treatments will likely be dependent on individual patient pre-treatment microbiome composition. Therefore, concurrent research in microbiome diagnostics and the perspective of precision medicine are vital for the development of microbiota-directed therapeutics.

Ultimately, we are likely to see emergence of a variety of effective microbial therapeutics, which may be composed of individual microbial strains, defined microbial consortia, or FMT-based products. Success of their development will depend on understanding the roles that individual microbial strains and microbial consortia play in specific disease conditions, and careful evaluation of their short- and long-term harms and benefits. At present, FMT products may be the most effective treatments for restoration of decimated microbiota, and we can anticipate the identification of microbiome signatures for FMT products that may be optimal for specific disease indications. The intestinal microbiota is a new therapeutic frontier of medicine, and the scientific tools are already in place to move this field forward.

## Abbreviations

FAO: Food and Agriculture Organization
FDA: Food and Drug Administration
FMT: Fecal microbiota transplantation
WHO: World Health Organization
